# The Cerebrospinal Fluid Profile of Cholesterol Metabolites in Parkinson’s Disease and Their Association With Disease State and Clinical Features

**DOI:** 10.3389/fnagi.2021.685594

**Published:** 2021-08-30

**Authors:** William J. Griffiths, Jonas Abdel-Khalik, Sarah F. Moore, Ruwani S. Wijeyekoon, Peter J. Crick, Eylan Yutuc, Krista Farrell, David P. Breen, Caroline H. Williams-Gray, Spyridon Theofilopoulos, Ernest Arenas, Miles Trupp, Roger A. Barker, Yuqin Wang

**Affiliations:** ^1^Swansea University Medical School, ILS1 Building, Swansea, United Kingdom; ^2^Department of Clinical Neurosciences, John van Geest Centre for Brain Repair, University of Cambridge, Cambridge, United Kingdom; ^3^Centre for Clinical Brain Sciences, University of Edinburgh, Edinburgh, United Kingdom; ^4^Anne Rowling Regenerative Neurology Clinic, University of Edinburgh, Edinburgh, United Kingdom; ^5^Usher Institute of Population Health Sciences and Informatics, University of Edinburgh, Edinburgh, United Kingdom; ^6^Division of Molecular Neurobiology, Department of Medical Biochemistry and Biophysics, Karolinska Institutet, Stockholm, Sweden; ^7^Department of Clinical Science, Neurosciences, Umeå University, Umeå, Sweden; ^8^Wellcome-MRC Cambridge Stem Cell Institute, University of Cambridge, Cambridge, United Kingdom

**Keywords:** sterol, oxysterol, dihydroxycholesterol, bile acid biosynthesis, mass spectrometry

## Abstract

Disordered cholesterol metabolism is linked to neurodegeneration. In this study we investigated the profile of cholesterol metabolites found in the cerebrospinal fluid (CSF) of Parkinson’s disease (PD) patients. When adjustments were made for confounding variables of age and sex, 7α,(25R)26-dihydroxycholesterol and a second oxysterol 7α,x,y-trihydroxycholest-4-en-3-one (7α,x,y-triHCO), whose exact structure is unknown, were found to be significantly elevated in PD CSF. The likely location of the additional hydroxy groups on the second oxysterol are on the sterol side-chain. We found that CSF 7α-hydroxycholesterol levels correlated positively with depression in PD patients, while two presumptively identified cholestenoic acids correlated negatively with depression.

## Introduction

Parkinson’s disease (PD) is a chronic neurodegenerative disorder of the central nervous system (CNS) that presents with motor deficits, but which also has many non-motor features, including cognitive and neuropsychiatric problems. In PD, the core motor features result mainly from a loss of dopaminergic neurons in the substantia nigra of the midbrain and their projection to the striatum, but more widespread pathology in subcortical and cortical regions, and even outside the CNS, underlies many of the non-motor features.

**FIGURE 1:**
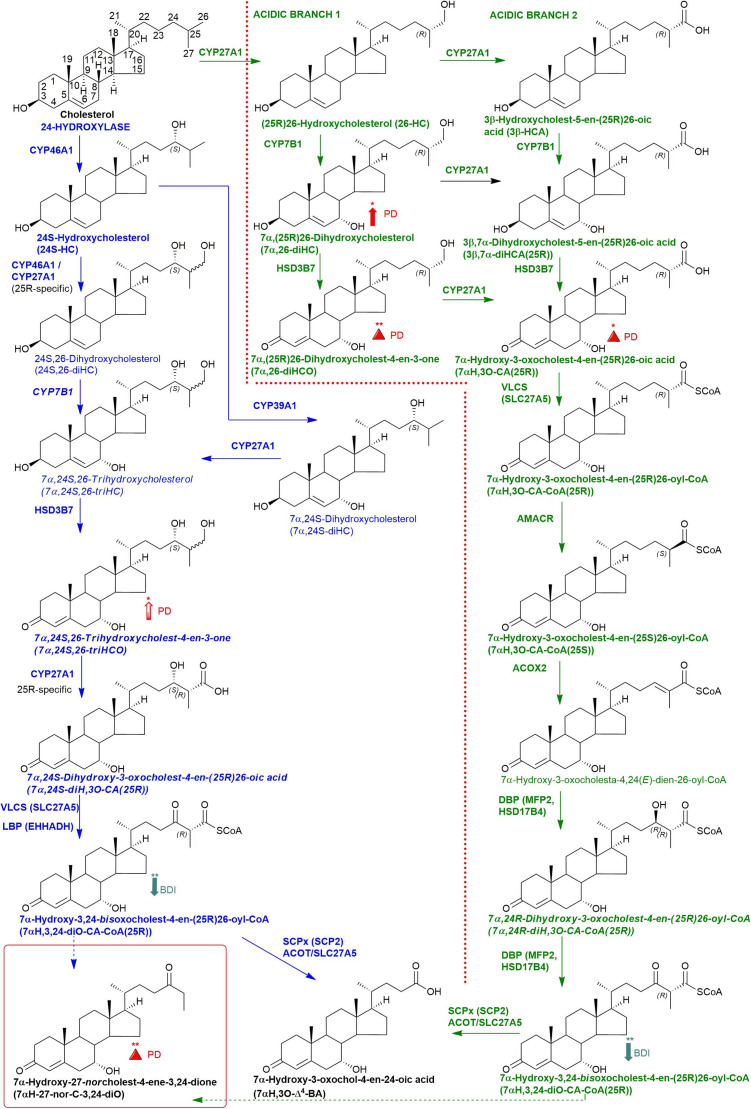
Abbreviated versions of the cerebral 24-hydroxylase (left) and acidic (right) pathways of bile acid biosynthesis. Enzymes, metabolites, and reactions of the 24-hydroxylase pathway are indicated in blue, those of the acidic pathway are in green. Enzymes/genes expressed in the brain, and metabolites observed, in CSF are in bold. CoA intermediates are observed as the unconjugated acids in CSF. *Italics* indicate that the named structure is one of a number of possibilities. The broken arrows indicate a reaction leading to elimination of C-27. Thick coloured arrows pointing upwards or downwards indicate significant positive or negative correlations even when the confounding variables are considered. Red triangles indicate significance ignoring confounding variables, in at least one of the two studies. The full stereochemistry and numbering system for cholesterol is indicated. Abbreviated structures are shown for other sterols ignoring ring-stereochemistry.

About 25% of total body cholesterol is found in the brain (Dietschy and Turley, 2004), and dysregulated cholesterol metabolism is linked to PD as it is to a number of other neurodegenerative conditions (Leoni et al., 2004; Leoni and Caccia, 2011; Bjorkhem et al., 2013, 2018). Cholesterol will not pass the blood brain barrier (BBB), and cannot be imported from the circulation, so essentially all brain cholesterol is synthesised *in situ*. Excess cholesterol is removed from the brain by the neuron-specific cytochrome P450 (CYP) 46A1- catalyzed metabolism to 24S-hydroxycholesterol (24S-HC, see Figure 1 for structure), which by virtue of its side-chain hydroxy group can cross the BBB and enter the circulation (Lutjohann et al., 1996). While 24S-HC exits the brain, (25R)26-hydroxycholesterol (26-HC), also known by the non-systematic name 27-hydroxycholesterol (Fakheri and Javitt, 2012), enters the brain from the circulation (Heverin et al., 2005), and is metabolised by CYP7B1, CYP27A1 and hydroxysteroid dehydrogenase (HSD) 3B7 to 7α-hydroxy-3-oxocholest-4-en-(25R)26-oic acid [7αH,3O-CA(25R), Figure 1] which is exported from the brain to the circulation and is also found in cerebrospinal fluid (CSF) (Meaney et al., 2007; Ogundare et al., 2010). Plasma and CSF levels of 24S-HC have been suggested as biomarkers for neurodegenerative disorders (Leoni et al., 2004), and while the prevailing evidence suggests that 24S-HC in plasma does not provide a diagnostic marker for PD (Bjorkhem et al., 2013, 2018), some data suggests that there may be a statistically significant elevation of 24S-HC in the CSF of PD patients (Bjorkhem et al., 2018).

Currently, oxysterols in the circulation and in CSF are almost exclusively analysed by mass spectrometry (MS) either in combination with gas chromatography (GC) (i.e., GC-MS) or with liquid chromatography (LC) (i.e., LC-MS) (Leoni et al., 2004; Griffiths et al., 2013). Most studies of oxysterols in CSF are not performed on the “free” non-esterified molecules which are exported from brain but on a combination of esterified and non-esterified molecules (Leoni et al., 2004; Bjorkhem et al., 2018). This is for practical reasons as the non-esterified molecules make up only a small proportion of the total as they become esterified by lecithin–cholesterol acyltransferase (LCAT) in lipoprotein particles within the CSF. However, there is value in analysing the non-esterified molecules alone as these are the precise forms exported from brain.

**FIGURE 2:**
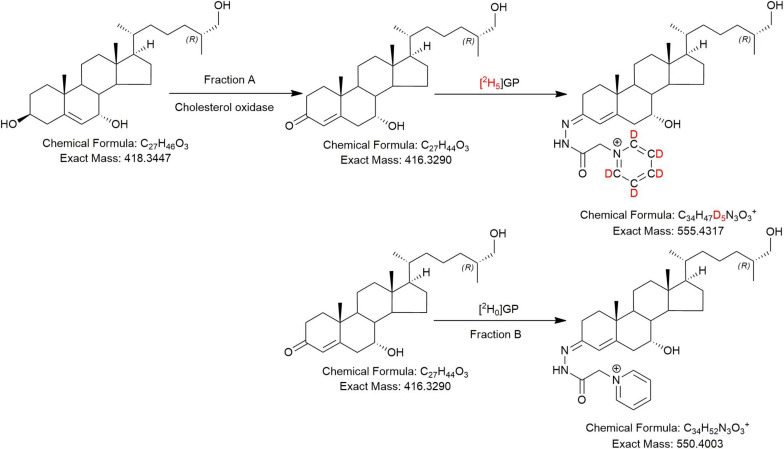
The EADSA derivatisation method. Samples are split into two equal fractions A and B. A-fractions are treated with cholesterol oxidase enzyme and [^2^H_5_]GP, B-fractions are treated with [^2^H_0_]GP in the absence of cholesterol oxidase. LC-MS(MS^3^) analysis of fraction-A reveals compounds with a 3β-hydroxy-5-ene structure plus those with a native 3-oxo-4-ene structure, while analysis of fraction-B reveals native 3-oxo-4-ene compounds only. Deconvolution of data from A and B allows quantification of 3β-hydroxy-5-ene compounds while data in B allows quantification of native 3-oxo-4-ene compounds (and 7-oxo-5-ene).

In the current study, we analysed “free” non-esterified oxysterols (including cholestenoic acids) in the CSF of PD patients and healthy controls with an aim of identifying metabolites or pathways linked to PD. To achieve the necessary sensitivity, we adopted a two-step derivatisation approach named “enzyme-assisted derivatisation for sterol analysis” (EADSA) in combination with LC-MS (Figure 2; Crick et al., 2015, 2017). Although we did not find a statistical increase in 24S-HC in CSF from PD patients compared to controls, we did find an increase in 7α,(25R)26-dihydroxycholesterol (7α,26-diHC), an intermediate in the pathway from 26-HC to 7αH,3O-CA(25R) (Figure 1). In addition, we found a positive correlation between the CSF concentration of 7α-hydroxycholesterol (7α-HC) and scores on the Beck Depression Inventory (BDI), which is a rating scale commonly used to assess depression in PD. Interestingly there were negative correlations between the presumptively identified cholestenoic acids, 7α-hydroxy-3,24-*bis*oxocholest-4-en-26-oic acid (7αH-3,24-diO-CA) and 7α,12α-dihydroxy-3-oxocholeste-4-en-26-oic acid (7α,12α-diH,3O-CA), and scores on the BDI but not other clinical measures. This work highlights the potential clinical significance of the bile acid biosynthesis pathway in PD and defines a methodology that can be used to measure the pathway intermediates within a clinical laboratory setting.

## Materials and Methods

### Subjects and Sample Collection

This work was designed in two studies: Study 1 primarily focused on oxysterol and cholestenoic acid identification while Study 2 focused on their quantitation and relationship with a range of PD relevant clinical measures. All patients were recruited from the Parkinson’s Disease Research Clinic at the John van Geest Centre for Brain Repair in Cambridge. The study was approved by the Cambridgeshire 2 Research Ethics Committee (Ref. 08/H0308/331) and written informed consent was obtained from all participants. Controls for Study 2 were carers of patients with PD with no known neurological disease, or patients attending Addenbrooke’s Hospital NHS Neurology clinics for a lumbar puncture to investigate other symptoms (such as headache), but with no known neurodegenerative disease.

Lumbar punctures were performed using an aseptic technique as per standard clinical guidelines. 2–5 mL of CSF was collected. The CSF was centrifuged at 2,000–3,000 g for 15 min and the supernatant was stored at −80°C prior to analysis.

Standard demographic data was collected along with assessments of disease severity including the Movement Disorder Society-Unified Parkinson’s Disease Rating Scale (MDS-UPDRS); neuropsychological assessments including the Addenbrooke’s Cognitive Examination Revised (ACE-R) and semantic fluency and assessment of depression using the BDI.

### LC-MS

The LC-MS method is described in Crick et al. (2015, 2017); it incorporated EADSA (Figure 2) to enhance sensitivity and specificity, reversed-phase chromatography to separate diastereoisomers, accurate mass measurement (<5 ppm) at high-resolution (30,000 in Study 1, 120,000 in Study 2, both at *m/z* 400) and multistage fragmentation (MS^n^) for structure determination. Quantification was performed against added isotope-labelled standards. In Study 1 quantification was against [25,26,26,26,27,27,27-^2^H_7_]24R/S-hydroxycholesterol ([^2^H_7_]24R/S-HC) which has been shown to be an adequate surrogate for side-chain oxysterols and cholestenoic acids (Crick et al., 2015). For Study 2, the additional standard [26,26,26,27,27,27-^2^H_6_]7α,25-dihydroxycholesterol ([^2^H_6_]7α,25-diHC) was included to allow quantification of 7α,25-dihydroxycholesterol (7α,25-diHC) and 7α,26-diHC and their 3-oxo analogues (Crick et al., 2017).

### Patient Data and Statistical Analysis

#### Study 1

This study was designed to allow for the identification of oxysterols including cholestenoic acids in CSF from PD patients. CSF from 18 PD patients was analysed and compared to a historical data set (Crick et al., 2017) of 18 control CSF samples from people without neurodegenerative conditions. Statistical significance was determined by the Mann-Whitney Test and confounding variables of sex and age were not considered.

#### Study 2

CSF samples from a separate cohort of PD patients and controls were analysed for oxysterols, including cholestenoic acids, and their relationship with a range of standard clinical measures was investigated (Table 1) in a cross-sectional study. Statistical analysis was performed using Stata software (Stata Statistical Software: Release 14. StataCorp LP, College Station, TX). Pairwise correlations with oxysterol data were performed for continuous demographic and clinical variables. Those correlations with *P* < 0.05 were entered into multiple regression analyses with the oxysterol as the dependent variable and inclusion of relevant confounding variables. For motor scores and BDI, these confounding variables were age, gender and years from onset of disease. For cognitive variables BDI score was also included as a potential confounder. For categorical variables ANOVA was performed, again adjusting for potential confounding variables as above. For clinical scores, data was only used if it had been generated within1 year of the lumbar puncture.

**TABLE 1:** Study 2 participant demographics.

Factor (Mean ± SD)	Patients (*n* = 37)	Controls (*n* = 5)
Age (y)	65.10 ± 8.24	63.60 ± 8.08
Gender (% Male)	45.94	40.00
Years from disease onset	3.98 ± 5.67	
MDS-UPDRS motor score (in the “ON” state)	32.82 ± 11.78	
ACE-R	90.70 ± 9.46	
Semantic fluency	24.8 ± 7.40	
BDI	9.62 ± 7.02	

## Results

### Study 1—Identification of Oxysterol and Cholestenoic Acids in CSF

**FIGURE 3:**
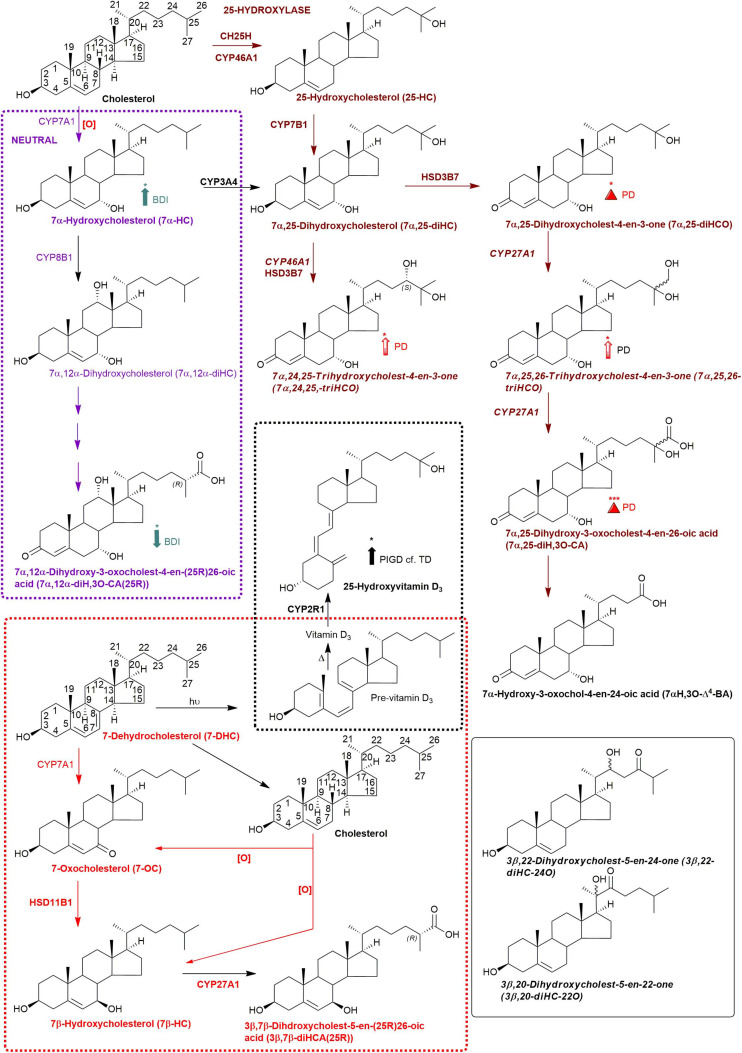
Abbreviated versions of the early steps in the neutral (left, in purple) and the cerebral 25-hydroxylase (right, in brown) pathways of bile acid biosynthesis. Pathway from 7-dehydrocholesterol and cholesterol to 3β,7β-diHCA(25R) are also shown as is the path to 25-hydroxyvitamin D_3_ in red and black dashed boxes, respectively. Enzymes, metabolites, and reactions of the neutral pathway are in purple, those of the 25-hydroxylase pathway are in brown, while those generating 3β,7β-diHCA(25R) are in red. Enzymes/genes expressed in brain, and metabolites observed in CSF are in bold. *Italics* indicate that the named structure is one of a number of possibilities. Enzymes in *italics* are postulated catalysts. [O] indicates oxidation *via* non-enzymatic mechanism. Thick coloured arrows pointing upwards or downwards indicate significant positive or negative correlations even when the confounding variables are considered. Red triangles indicate significance ignoring confounding variables, in at least one of the two studies. The full stereochemistry and numbering system for cholesterol and 7-DHC is indicated. Abbreviated structures are shown for other sterols ignoring ring-stereochemistry.

**FIGURE 4:**
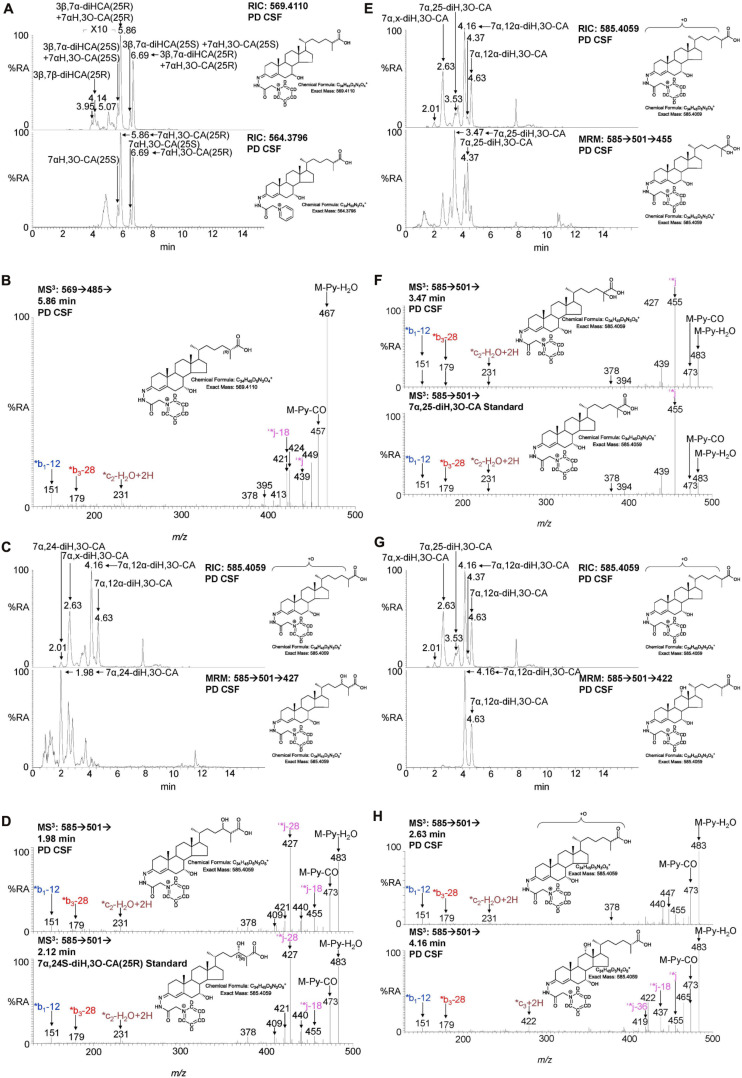
LC-MS(MS^n^) analysis of cholestenoic acids found in CSF. **(A)** RIC of *m/z* 569.4110 ± 5 ppm revealing 3β,7β-diHCA and 3β,7α-diHCA (upper panel) and of 564.3796 ± 5 ppm revealing 7αH,3O-CA (lower panel). Each acid is present as two epimers, and each epimer gives *syn* and *anti* conformers of the GP-derivative. **(B)** MS^3^ (M^+^ → M^+^-Py →) spectrum of 3β,7α-diHCA(25R). **(C,E,G)** RIC of *m/z* 585.4059 ± 5 ppm revealing dihydroxy-3-oxocholestenoic acids (upper panels) and MRM-like chromatogram 585.4 → 501.3 → 427 highlighting 7α,24-diH,3O-CA (lower panel C), 585.4 → 501.3 → 455 highlighting 7α,25-diH,3O-CA (lower panel E) and 585.4 → 501.3 → 422 highlighting 7α,12α-diH,3O-CA (lower panel G). MS^3^ (M^+^ → M^+^-Py →) spectra of **(D)** 7α,24-diH,3O-CA in CSF (upper panel) and 7α,24S-diH,3O-CA(25R) authentic standard (lower panel), **(F)** 7α,25-diH,3O-CA in CSF (upper panel) and 7α,25-diH,3O-CA authentic standard (lower panel), and **(H)** 7α,x-diH,3O-CA (upper panel) and 7α,12α-diH,3O-CA (lower panel) in CSF.

Initial studies were performed on 18 CSF samples from early-mid stage PD patients [72% male, mean (standard deviation, SD) age = 69 (7) years, disease duration = 4 (4) years, MDS-UPDRS motor score on treatment = 31(12), ACE-R = 89 (8), BDI = 6 (6)] with the aim of identifying non-esterified oxysterols present in the CSF. The oxysterols identified in this first study are listed in Table 2. In addition to the expected monohydroxycholesterols, 24S-HC, 25-hydroxycholesterol (25-HC) and 26-HC, we identified (but did not quantify) the dihydroxycholesterols 7α,25-diHC and 7α,26-diHC and their dihydroxycholest-4-en-3-ones, i.e., 7α,25-dihydroxycholest-4-en-3-one (7α,25-diHCO) and 7α,(25R)26-dihydroxycholest-4-en-3-one (7α,26-diHCO, Figures 1, 3). In addition, we identified and approximately quantified the cholestenoic acids, 3β-hydroxycholest-5-en-(25R)26-oic acid (3β-HCA), and the 25R- and 25S-diastereoisomers of 3β,7β-dihydroxycholest-5-en-26-oic (3β,7β-diHCA), of 3β,7α-dihydroxycholest-5-en-26-oic (3β,7α-diHCA) and of 7αH,3O-CA (Figures 1, 3, 4A,B), as well as uncovering a series of dihydroxy-3-oxocholest-4-enoic acids (diH,3O-CA, Figures 4C–H). For this initial study, we did not have access to CSF samples from controls but compared the data from our PD patients to control data generated in a prior study (Crick et al., 2017).

**TABLE 2:** Oxysterols in CSF of PD patients and controls.

Fraction A	Fraction B	Sterol systematic name (common name)	Abbreviation	Study 1					Study 2					Note
*m/z*	*m/z*			ng/mL					ng/mL					
					
				PD		Control		Significance	PD		Control		Significance	
					
				Mean	SD	Mean	SD	PD vs Control	Mean	SD	Mean	SD	PD vs Control	
527.3640	522.3326	7α-Hydroxy-3-oxochol-4-en-24-oic acid	7αH,3O-Δ^4^-BA	**0.672**	0.246	**0.708**	0.237	NS	**0.675**	0.167	**0.551**	0.081	NS	
539.4004	534.369	7α-Hydroxy-27-nor-cholest-4-ene-3,24-dione	7αH-27-nor-C-3,24-diO	**0.387**	0.162	**0.245**	0.143	**	**0.698**	0.194	**0.638**	0.025	NS	1
539.4368	NA	Cholest-5-ene-3β,24S-diol (24S-hydroxycholesterol)	24S-HC	**0.050**	0.022	**0.045**	0.019	NS	**0.015**	0.009	**0.008**	0.004	NS	
539.4368	NA	Cholest-5-ene-3β,25-diol (25-hydroxycholesterol)	25-HC	**0.028**	0.028	**0.030**	0.019	NS	**0.016**	0.015	**0.012**	0.005	NS	
539.4368	NA	Cholest-5-ene-3β,(25R)26-diol ((25R),26-Hydroxycholesterol)	26-HC	**0.113**	0.064	**0.100**	0.028	NS	**0.093**	0.053	**0.064**	0.017	NS	
539.4368	NA	Cholest-5-ene-3β,7β-diol (7β-Hydroxycholesterol)	7β-HC	**0.056**	0.066	**0.036**	0.027	NS	**0.181**	0.474	**0.082**	0.034	NS	2
539.4368	534.4054	3β-Hydroxycholest-5-en-7-one (7-Oxocholesterol)	7-OC	**0.601**	0.513	**0.378**	0.225	NS	**0.671**	0.671	**0.731**	0.540	NS	2
539.4368	NA	Cholest-5-ene-3β,7α-diol (7α-Hydroxycholesterol)	7α-HC	**0.063**	0.067	**0.039**	0.032	NS	**0.091**	0.118	**0.056**	0.027	NS	2
539.4368	NA	Cholest-5-ene-3β,6β-diol (6β-Hydroxycholesterol)	6β-HC	**0.345**	0.234	**0.280**	0.312	NS	**0.918**	1.289	**0.593**	0.141	NS	3
537.4212	NA	9,10-Secocholesta-5,7,10-triene-3β,25-diol (25-hydroxyvitamin D_3_)	25-D_3_	NM	NM	NM	NM	NA	**0.171**	0.095	**0.140**	0.057	NS	
551.4004	546.369	3-Oxocholesta-4,6-dien-26-oic acid	–	**2.654**	2.426	**1.546**	0.468	NS	**1.461**	0.496	**1.154**	0.335	NS	4
551.4004	NA	3β-Hydroxycholesta-5,7-dien-26-oic acid	–	**0.318**	0.334	**0.079**	0.099	**	**0.142**	0.143	**0.043**	0.038	NS	5
553.4161	NA	3β,x-Dihydroxycholest-5-en-y-one	3β,x-diHC-yO	NM	NM	NM	NM	NA	**0.050**	0.036	**0.066**	0.028	NS	6
553.4161	NA	3β-Hydroxycholest-5-en-(25R)26-oic acid	3β-HCA	**1.073**	0.793	**0.959**	0.416	NS	**1.210**	0.557	**0.899**	0.287	NS	
555.4317	550.4003	7α,25-Dihydroxycholest-4-en-3-one	7α,25-diHCO	NM	NM	NM	NM	NA	**0.009**	0.005	**0.006**	0.001	*	
555.4317	NA	Cholest-5-ene-3β,7α,25-triol (7α,25-Dihydroxycholesterol)	7α,25-diHC	NM	NM	NM	NM	NA	**0.006**	0.005	**0.006**	0.004	NS	
555.4317	550.4003	7α,(25R)26-Dihydroxycholest-4-en-3-one	7α,26-diHCO	NM	NM	NM	NM	NA	**0.009**	0.004	**0.005**	0.001	**	
555.4317	NA	Cholest-5-ene-3β,7α,(25R)26-triol (7α,(25R)26-Dihydroxycholesterol)	7α,26-diHC	NM	NM	NM	NM	NA	**0.005**	0.002	**0.002**	0.002	*	
567.3953	562.3639	x-Hydroxy-3-oxocholesta-4,6-dien-26-oic acid	–	**0.190**	0.169	**0.112**	0.041	NS	**0.453**	0.143	**0.361**	0.119	NS	
567.3953	562.3639	x-Hydroxy-3-oxocholesta-4,6-dien-26-oic acid	–	**0.100**	0.090	**0.069**	0.036	NS	NM	NM	NM	NM	NA	
569.4110	NA	3β,7β-Dihydroxycholest-5-en-26-oic acid	3β,7β-diHCA	**0.455**	0.212	**0.403**	0.190	NS	**0.506**	0.169	**0.406**	0.104	NS	7
569.4110	NA	3β,x,y-Trihydroxycholest-5-en-z-one	3β,x,y-triHC-zO	**0.228**	0.122	**0.147**	0.067	*	**0.172**	0.061	**0.127**	0.036	NS	8
569.4110	564.3796	7α-Hydroxy-3-oxocholest-4-en-26-oic acid	7αH,3O-CA	**22.728**	11.445	**15.851**	4.305	*	**21.198**	6.292	**17.731**	3.983	NS	7,9
569.4110	NA	3β,7α-Dihydroxycholest-5-en-26-oic acid	3β,7α-diHCA	**3.235**	3.308	**2.042**	1.577	NS	**3.808**	2.258	**1.785**	1.575	NS	7,10
571.4266	566.3952	7α,x,y-Trihydroxycholest-4-en-3-one	7α,x,y-triHCO	**0.198**	0.258	**0.286**	0.116	NS	**0.116**	0.062	**0.068**	0.013	*	11
583.3903	578.3589	7α-Hydroxy-3,24-*bis*oxocholest-4-en-26-oic acid	7αH,3,24-diO-CA	**0.236**	0.082	**0.208**	0.057	NS	**0.285**	0.094	**0.227**	0.065	NS	12
585.4059	580.3745	7α,24-Dihydroxy-3-oxocholest-4-en-26-oic acid	7α,24-diH,3O-CA	NM	NM	NM	NM	NA	**0.312**	0.080	**0.251**	0.021	NS	
585.4059	580.3745	7α,x-Dihydroxy-3-oxocholest-4-en-26-oic acid	7α,x-diH,3O-CA	**5.212**	1.737	**2.938**	0.887	***	**5.938**	1.522	**5.038**	1.314	NS	13
585.4059	580.3745	7α,25-Dihydroxy-3-oxocholest-4-en-26-oic acid	7α,25-diH,3O-CA	**1.306**	0.472	**0.715**	0.224	***	**1.634**	0.458	**1.353**	0.213	NS	
585.4059	580.3745	7α,12α-Dihydroxy-3-oxocholest-4-en-26-oic acid	7α,12α-diH,3O-CA	NM	NM	NM	NM	NA	**1.100**	1.176	**1.157**	0.600	NS	
601.4008	596.3694	Trihydroxy-3-oxocholest-4-en-26-oic acid	triH,3O-CA	**0.021**	0.063	**0.077**	0.041	*	NM	NM	NM	NM	NA	
		TOTAL 7α-Hydroxy-3-oxocholest-4-en-26-oic acid	7αH,3O-CA	**25.383**	13.206	**17.396**	4.628	*	**22.659**	6.745	**18.886**	4.312	NS	14
		TOTAL 3β,7α-Dihydroxycholest-5-en-26-oic acid	3β,7α-diHCA	**3.553**	3.477	**2.121**	1.648	NS	**3.950**	2.384	**1.828**	1.606	NS	15

*P < 0.05, **P < 0.01, ***P < 0.001 determined using Mann–Whitney Test. NA, not applicable. As a visual aid concentrations are written in bold. 1. Decarboxylation product of 7α-hydroxy-3,24-bisoxocholest-4-en-26-oic acid. 2. May be formed enzymatically or by in vivo or ex vivo autoxidation. 3. 6β-HC is the dehydration product of cholestane-3β,5α,6β-triol, formed from 5,6-epoxycholesterol. 4. Dehydration product of 7αH,3O-CA. 5. Dehydration product of 3β,7-diHCA. 6. x and y probably correspond to 22 and 24 or 20 and 22, based on MS^3^ spectra. 7. 25R and 25S epimers measured in combination. 8. x, y and z probably 22, 25 and 24. 9. Some dehydration of 7αH,3O-CA (see 4). 10. Some dehydration of 3β,7-diHCA (see 5). 11. x any y probably 24,25, 24,26, or 25,26. 12. Undergoes decarboxylation to 7α-hydroxy-27-nor-cholest-4-ene-3,24-dione (see 1). 13. x is probably on the side-chain. 14. Total 7α-hydroxy-3-oxocholest-4-en-26-oic acid is a combination of molecule and its dehydrated analogue. 15. Total 3β,7α-dihydroxycholest-5-en-26-oic acid is a combination of molecule and its dehydrated analogue.

**FIGURE 5:**
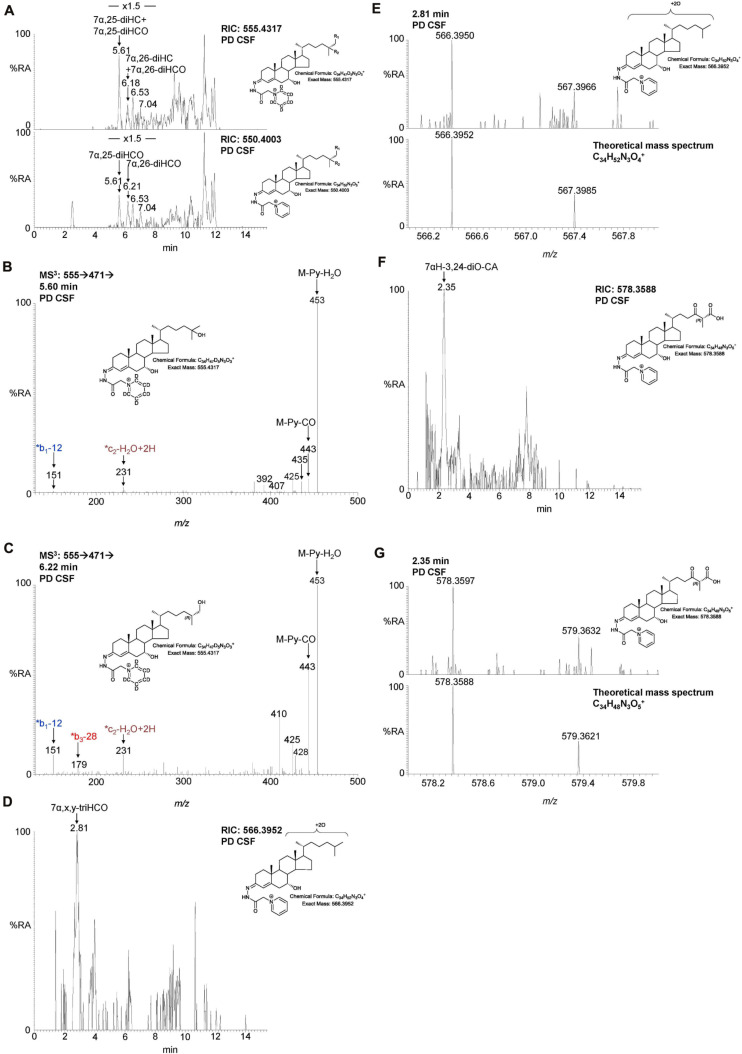
LC-MS(MS^*n*^) analysis of dihydroxycholesterols, dihydroxycholestenones, trihydroxycholestenones, and hydroxy*bis*oxocholestenoic acid in CSF. **(A)** RIC of 555.4317 ± 5 ppm (upper panel) and 550.4003 ± 5 ppm (lower panel) revealing 7α,25-diHC, 7α,26-diHC, 7α,25-diHCO, and 7α,26-diHCO. MS^3^ (M^+^ → M^+^-Py→) spectra revealing **(B)** 7α,25-diHC and **(C)** 7α,26-diHC. **(D)** RIC of 566.3952 ± 3 ppm corresponding to 7α,x,y-triHCO. **(E)** High resolution (100,000) mass spectrum (upper panel) and theoretical mass spectrum (lower panel) of 7α,x,y-triHCO. **(F)** RIC of 578.3588 ± 3 ppm corresponding to 7αH-3,24-diO-CA. **(G)** High resolution (100,000) mass spectrum (upper panel) and theoretical mass spectrum (lower panel) of 7αH,3,24-diO-CA.

We have previously shown that the acidic pathway of bile acid biosynthesis is at least partially active in the brain (Ogundare et al., 2010). This pathway has two branches which start with (25R)26-hydroxylation and (25R)26-carboxylation of cholesterol by CYP27A1 to give 26-HC and 3β-HCA, respectively (Figure 1). 26-HC may be derived from cholesterol in the brain or imported from the circulation (Heverin et al., 2005). These two metabolites are 7α-hydroxylated by CYP7B1 to give 7α,26-diHC and 3β,7α-diHCA(25R), respectively (Figure 1) and after oxidation at C-3 and Δ^5^ to Δ^4^ isomerisation the branches converge at 7αH,3O-CA(25R). We observed each of these metabolites in the CSF and notably the concentration of 7αH,3O-CA was specifically elevated in PD CSF (*P* < 0.05, Table 2). It should be noted that both 25R- and 25S-diastereoisomers of 3β,7α-diHCA and 7αH,3O-CA are present in CSF, where the 25R-epimer dominates, however, as the epimers are not fully resolved chromatographically we have measured the two in combination (Figure 4A). In the next steps of the acidic pathway 7αH,3O-CA(25R) becomes converted to the CoA thioester and through multiple steps to 7α,24R-dihydroxy-3-oxocholest-4-en-(25R)26-oyl-CoA (7α,24R-diH,3O-CA(25R)-CoA, Figure 1; Ferdinandusse et al., 2009; Autio et al., 2014; Griffiths and Wang, 2020), and by generating the appropriate reconstructed ion chromatogram (RIC), we were able to identify a number of chromatographic peaks potentially corresponding to the acid form of this structure (Figure 4C). Notably, in CSF and plasma we do not find CoA thioesters but rather the free acids. The CoA thioester of 7α,24R-diH,3O-CA(25R) is a key intermediate in side-chain shortening of C_27_ to C_24_ bile acids, becoming oxidised to 7α-hydroxy-3,24-*bis*oxocholest-4-en-(25R)26-oyl-CoA (7αH,3,24-diO-CA(25R)-CoA, Figure 5F). This metabolite is not fully stable in our methodology partially eliminating the C-26 group to give 7α-hydroxy-27-*nor*cholest-4-ene-3,24-dione (7αH-27-nor-C-3,24-diO, see Supplementary Figure 1) (Ogundare et al., 2010). We found 7αH-27-nor-C-3,24-diO to be elevated significantly in the CSF from PD patients (*P* < 0.01). In combination, this initial study suggests the acidic pathway is upregulated in the CNS of PD patients.

We were also able to partially identify a number of other oxysterols in the CSF based on retention time, accurate mass and MS^3^ spectra, but in the absence of authentic standards, definitive identifications were not made. These partial identifications include 3β,x-dihydroxycholest-5-en-y-one (3β,x-diHC-yO) where x and y may be 22 and 24, or 20 and 22, and 7α,x,y-trihydroxycholest-4-en-3-one (7α,x,y-triHCO, Figure 5D) where x and y may be 24, 25, or 26 (*italic* compound names in Figures 1, 3).

We next performed multivariate analysis on the data from Study 1 using SIMCA software and an orthogonal projection to latent structures discriminant analysis (OPLS-DA) and this yielded a robust model separating PD from controls (Supplementary Figure 2, *Q*2 = 0.68, ANOVA = 3.2e-7 for cross-validated model), suggesting a cluster of cholesterol metabolites as candidate biomarkers for PD. This data should be treated with caution as the patient and control data were reordered at different times and for samples collected from different hospitals in different countries. Nevertheless, metabolites significant in the univariate analysis (Table 2) were important in driving the separation in the multivariate model.

### Study 2—CSF Oxysterols, Disease Status, and Clinical Measures of Disease

In this second study, data from 37 PD cases was compared to 5 age-matched controls. Relevant demographic and clinical variables are shown in Table 1. Internal standards were also included allowing for the quantification of 7α,26-diHC and 7α,26-diHCO (Figure 5) of the acidic pathway and also 7α,25-diHC and 7α,25-diHCO. The availability of samples from matched controls collected from the same geographical area (albeit in lower numbers than the patients) and the recording of LC-MS data in a single study allowed us to perform a deeper interrogation of the data than in Study 1. However, the number of control samples was limited and therefore PD vs. control comparisons need to be interpreted with caution.

#### 7α,26-diHC Is Elevated in PD CSF

**FIGURE 6:**
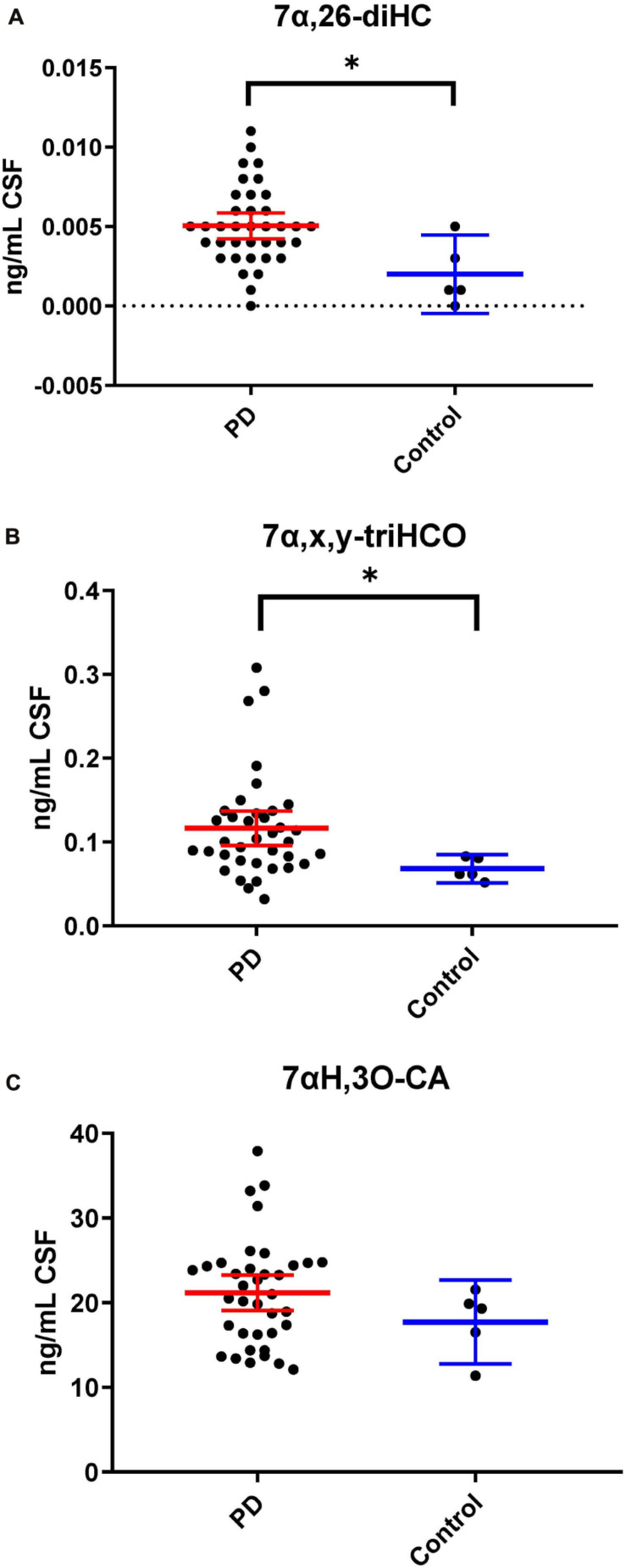
7α,26-diHC and 7α,x,y-triHCO are elevated in CSF from PD patients. **(A)** 7α,26-diHC. **(B)** 7α,x,y-triHCO. **(C)** 7αH,3O-CA is also elevated numerically but the significance is lost when confounding variables of age and sex are considered. Data from Study 2.

Following adjustment for the confounding variables of age and sex, 7α,26-diHC and a second oxysterol 7α,x,y-triHCO whose exact structure is unknown were found to be significantly elevated in PD CSF (Figures 6A,B). Based on accurate mass measurement, MS^3^ fragmentation and retention time 7α,x,y-triHCO is likely to be 7α,24,25-triHCO, 7α,24,26-triHCO or 7α,25,26-triHCO (the uncertainty of structure is indicated by italicised nomenclature in Figures 1, 3). Notably, 7α,26-diHC is an intermediate of the acidic pathway of bile acid biosynthesis (Figure 1). It was identified in Study 1 but not quantified due to an absence of an appropriate internal standard. Numerically, as in Study 1, 7αH,3O-CA (Figure 6C), 7αH-27-nor-C-3,24-diO (and its chemically unstable precursor 7αH,3,24-diO-CA) were elevated in PD CSF in Study 2, but not to a level of statistical significance (Table 2).

During the intervening period between conducting Study 1 and 2, we were able to purchase the trihydroxycholestenoic acids 3β,7α,24S-trihydroxycholest-5-en-(25R)26-oic (3β,7α,24S-triHCA(25R)) and 3β,7α,25-trihydroxycholest-5-en-26-oic (3β,7α,25-triHCA) acids from Avanti Polar Lipids Inc., which are easily converted in the laboratory to 7α,24S-dihydroxy-3-oxocholest-4-en-(25R)26-oic (7α,24S-diH,3O-CA(25R)) and 7α,25-dihydroxy-3-oxocholest-4-en-26-oic (7α,25-diH,3O-CA) acids, respectively, by treatment with cholesterol oxidase enzyme (Abdel-Khalik et al., 2018). This allowed us to identify and approximately quantify both acids in the CSF from PD patients and controls (Figures 4C–F). In the absence of 24S,25S, 24R,25R and 24R,25S diastereoisomers, it was not possible to define the exact stereochemistry for 7α,24-diH,3O-CA, and it may be 24S,25R, 24R,25R, 24S,25S or a mixture of all depending on the pathway(s) of biosynthesis (Figure 1; Autio et al., 2014). We were able to presumptively identify two other acids, as 7α,12α-dihydroxy-3-oxocholest-4-en-(25R)26-oic acid (7α,12α-diH,3O-CA) and 7α,x-dihydroxy-3-oxocholest-4-en-26-oic acid (7α,x-diH,3O-CA) based on retention time, accurate mass and MS^3^ spectra (Figures 4G,H). The location of the second hydroxy group in 7α,x-diH,3O-CA is probably on the side-chain.

Combining data from Study 1 and Study 2, we have found that the acidic pathway of bile acid biosynthesis is upregulated in the CNS of PD patients (Figure 1).

#### Correlations With Clinical Data

**FIGURE 7:**
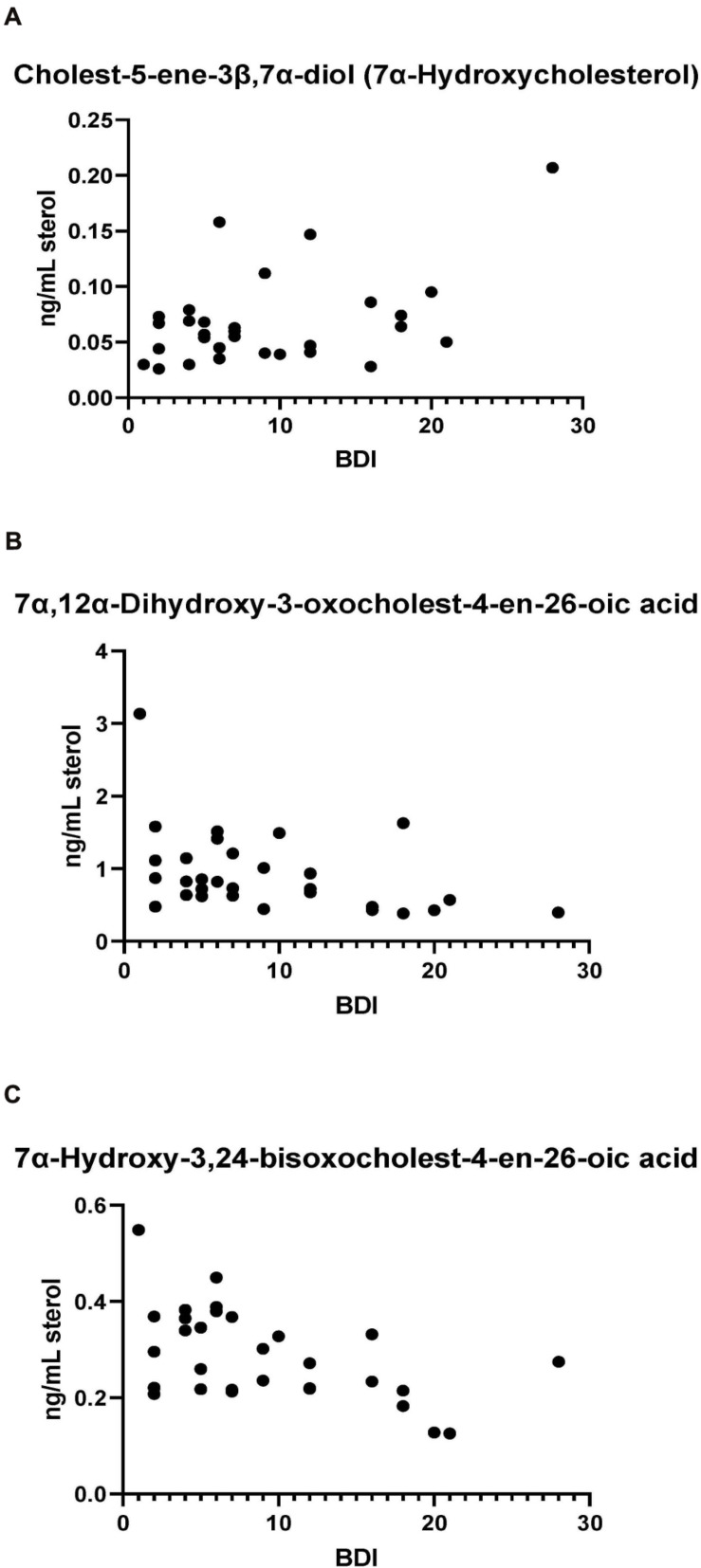
**(A)** 7α-Hydroxycholesterol correlates positively with Beck Depression Inventory (BDI) scores while presumptively identified acids, **(B)** 7α,12α-dihydroxy-3-oxochelest-4-en-26-oic, and **(C)** 7α-hydroxy-3,24-*bis*oxocholest-4-en-26-oic acids show negative correlations with the BDI score.

Bivariate correlation analyses between each PD CSF oxysterol profile and relevant demographic and clinical variables (age, gender, disease duration, MDS-UPDRS motor score, ACE-R score, BDI score) were performed. Correlations of significance (at a level of *P* < 0.05) were found between PD CSF 24S-HC and disease duration (*r* = 0.354, *P* = 0.032), 7α-HC and BDI (*r* = 0.436, *P* = 0.023), 7αH-3,24-diO-CA and BDI (*r* = –0.527, *P* = 0.005) and 7α,12α-diH,3O-CA and BDI (*r* = –0.418, *P* = 0.030). Multivariate regression analysis with 24S-HC as the dependent variable and age and gender as relevant covariates did not confirm the relationship between 24S-HC and disease duration (Beta coefficient 0.313, *P* = 0.060). However, multivariate analyses did confirm the relationships between 7α-HC, 7αH-3,24-diO-CA, 7α,12α-diH,3O-CA, and BDI, with age, gender, and disease duration as relevant confounding covariates (7α-HC: Beta coefficient 0.449, *P* = 0.031; 7αH-3,24-diO-CA: Beta coefficient –0.510, *P* = 0.010; 7α,12α-diH,3O-CA: Beta coefficient –0.414, *p* = 0.042, see Figure 7). There were no statistically significant associations between any of the CSF oxysterols and motor measures [MDS-UPDRS motor score, motor phenotype (tremor dominant vs. postural instability subtype)] or cognitive measures (ACE-R, semantic fluency). However, 25-hydroxyvitamin D_3_, the precursor of bioactive 1α,25-dihydroxyvitamin D_3_, is elevated in CSF of patients with postural instability and gait disturbance (PIGD) compared to tremor dominant patients (TD, *P* = 0.04). Although the reason for this is not known, it may be the case that PIGD patients are more likely to be given calcium/vitamin D supplements because they are at risk of falls. Vitamin D_3_ is converted to 25-hydroxyvitamin D_3_ in the liver and is transported in the blood stream to the kidney where 1α,25-dihydroxyvitamin D_3_ is formed.

## Discussion

In an early study looking at total oxysterols (where esterified and non-esterified molecules were measured in combination) in the CSF of PD patients and controls, concentrations of 24S-HC and 26-HC were found to be elevated in about 10% of PD samples above a cut off defined as the control mean + 3 standard deviations (SD) (Bjorkhem et al., 2013). However, when considering all samples, statistically significant differences were lost. In a follow-on study, Bjorkhem et al. (2018) found a small (about 1.75 ng/mL cf. 1.4 ng/mL) but statistically significant (*p* < 0.05) increase in 24S-HC in PD CSF. In this second study the CSF concentration of 24S-HC was found to correlate with disease progression. These results were suggested to relate to the release of 24S-HC from a subtype of dying neurons in PD, leading to an increase in 24S-HC concentration in the CSF during disease progression (Bjorkhem et al., 2013, 2018). The explanation for the increase in the CSF content of 26-HC in a sub-set of PD patients was suggested to be a consequence of a defective BBB and excessive import of 26-HC from the circulation (Bjorkhem et al., 2013, 2018).

In our current studies, we have measured the biologically more relevant non-esterified molecules. We did not find a statistically significant increase in 24S-HC in CSF from PD patients in either study. 7α,26-diHC, one of the immediate down-stream metabolites of 26-HC (Figure 1), was increased in PD CSF following correction for age and sex (Figure 6A). Closer evaluation of the data sets in both Study 1 and Study 2 show that although not statistically significant when confounding variables are adjusted for, early metabolites in the acidic pathway of bile acid biosynthesis are elevated in the CSF from PD patients (Figure 1). This supports the suggestion of Bjorkhem et al. (2013, 2018) that a defective BBB may be responsible for distorting the oxysterol pattern in CSF of PD patients. An alternative explanation is that cholesterol released by dying cells in the PD brain is metabolised by CYP27A1, CYP7B1 and HSD3B7 and shunted into the bile acid biosynthesis pathway (Figure 1). Interestingly, a recent study has found an upregulation of bacteria responsible for secondary bile acid synthesis in the gastrointestinal tract of PD patients (Li et al., 2021), although how this may relate to CSF changes is not clear.

In brain, the origin of 26-HC may be cerebral or *via* import across the BBB (Heverin et al., 2005), however, there is strong evidence for its conversion to 7αH,3O-CA(25R) in the brain itself (Meaney et al., 2007; Ogundare et al., 2010). Importantly, the necessary enzymes, or their transcripts, for the conversion of 7αH,3O-CA(25R) to the C_24_ bile acid 7α-hydroxy-3-oxochol-4-en-24-oic acid (7αH,3O-Δ^4^-BA) are all expressed in human brain (see Figure 1; Uhlen et al., 2015; Baloni et al., 2020).

A major route for 24S-HC metabolism is by CYP39A1 catalyzed 7α-hydroxylation to 7α,24S-dihydroxycholesterol (7α,24S-diHC, Figure 1) in the liver and onward to bile acids (Russell, 2003; Griffiths and Wang, 2020). CYP39A1 is, however, also expressed in the cerebellum and at low levels in the midbrain (Uhlen et al., 2015), providing a potential route to bile acid biosynthesis from 24S-HC in the brain. Although we did not identify 7α,24S-diHC in human CSF we did find the down-stream metabolic product 7αH,3,24-diO-CA, and its decarboxylation product 7αH-27-nor-C-3,24-diO. It should, however, be noted that 7αH,3,24-diO-CA is also a member of the acidic pathway (Figure 1). Interestingly, 7α,x,y-triHCO, is elevated in the CSF of PD patients (Figure 6B), and if x and y are 24S- and 26-hydroxy groups, respectively, then this metabolite falls into the metabolic pathway originating from 24S-HC.

Cholesterol 7α-hydroxylase (CYP7A1) is not expressed in brain (Uhlen et al., 2015; Baloni et al., 2020), hence the presence of 7α-HC in CSF must be *via* the circulation or *via* non-enzymatic oxidation of cholesterol. 7α-HC represents the first member of the neutral pathway of bile acid biosynthesis (Russell, 2003), one of the branches of this pathway proceeds through 7α,12α-diH,3O-CA which is one of the acids we presumptively identify in CSF. CYP8B1 is the necessary sterol 12α-hydroxylase but has not been found in human brain (Uhlen et al., 2015; Baloni et al., 2020), suggesting that the origin of 7α,12α-diH,3O-CA is from the circulation. While the 7α,24- and 7α,25-dihydroxy acids found in CSF are barely detected in plasma, 7α,12α-diH,3O-CA is present at the ng/mL level (Abdel-Khalik et al., 2017). In combination this data argues for an extracerebral origin for 7α,12α-diH,3O-CA and its import into CSF from the circulation. In future studies we recommend that wherever possible plasma and CSF from the same PD patient should be analysed in parallel. This will support or refute the hypothesis that the origin of some oxysterols and cholestenoic acids found in CSF is from the circulation. Assessing the correlations for each analyte between the two media should give a good indication if the origin of the metabolite is extra- or intra-CNS. To investigate the possibility of blood contamination confounding the CSF data, a simple extension to the experimental protocol would be to record a direct infusion mass spectrum from a few μL of CSF to identify the presence or absence of haemoglobin. In the present study we did not perform such an analysis, but any contamination by blood can only be minimal as in all CSF samples 3β-HCA was only a minor oxysterol while it is the most abundant free oxysterol in plasma (Abdel-Khalik et al., 2017).

The levels of the oxysterols 7α-HC, 7αH-3,24-diO-CA, 7α,12α-diH,3O-CA were found to correlate with BDI score (Figure 7) in PD cases but not with other clinical measures. No previous studies have identified associations between oxysterols and depression in general. As these oxysterols are predominantly considered to originate from the circulation, this may suggest the involvement of biological processes of systemic origin in PD depression. Depression is known to be associated with markers of systemic inflammation (Miller and Raison, 2016), including in PD (Lindqvist et al., 2013), while oxysterols are known to contribute to inflammatory processes (Duc et al., 2019). Thus, systemic immune modulatory processes may be a potential linking factor mediating the observed relationship between oxysterol levels and depression. However, further studies in larger PD and matched control cohorts will be required to confirm and extend this association and its biological basis, as will measurement of these metabolites in PD plasma. A caveat to the link between oxysterols, inflammation and depression, is the lack of correlation between the major immunoregulatory oxysterols 25-HC and 7α,25-diHC with BDI score.

Interestingly, intermediates in the acidic pathway of bile acid biosynthesis have also been found to be elevated in people suffering from multiple sclerosis but not in those suffering from amyotrophic lateral sclerosis or Alzheimer’s disease (Abdel-Khalik et al., 2017; Crick et al., 2017; Griffiths et al., 2019), arguing against a link between a general mechanism for neurodegeneration and cerebral bile acid biosynthesis. Never-the-less, this work points to the potential value of measuring bile acid precursors in CSF in the clinical chemistry laboratory. Further studies with much greater numbers are required to assess the potential of CSF bile acid precursors as prognostic biomarkers or as lead compounds towards a PD therapeutic.

## Conclusion

In conclusion, despite the limitations mentioned around our control CSF sample collection, a number of interesting and novel observations have been made in our study. Our data suggests a cerebral upregulation of the acidic pathway of bile acid biosynthesis in PD. We have also identified a number of cholesterol metabolites whose CSF levels correlate with depression in PD. Further studies are planned utilising greater sample numbers to confirm or refute the current findings.

## Data Availability Statement

The raw data supporting the conclusions of this article will be made available by the authors, without undue reservation, to any qualified researcher.

## Ethics Statement

The studies involving human participants were reviewed and approved by the Cambridgeshire 2 Research Ethics Committee. The patients/participants provided their written informed consent to participate in this study.

## Author Contributions

WG, ST, EA, RB, and YW designed the study. JA-K, PC, EY, SM, RW, DB, and KF performed the study. CW-G, SM, and MT supervised and performed statistical analysis. All authors contributed to writing of the manuscript.

## Author Disclaimer

The views expressed are those of the author(s) and not necessarily those of the NIHR or the Department of Health and Social Care.

## Conflict of Interest

WG, PC, and YW were listed as inventors on the Swansea University patent “Kit and method for quantitative detection of steroids,” US9851368B2, licensed to Avanti Polar Lipids Inc., and Cayman Chemical Company by Swansea University. WG, JA-K, PC, EY, ST, EA, and YW were shareholders in CholesteniX Ltd. The remaining authors declare that the research was conducted in the absence of any commercial or financial relationships that could be construed as a potential conflict of interest.

## Publisher’s Note

All claims expressed in this article are solely those of the authors and do not necessarily represent those of their affiliated organizations, or those of the publisher, the editors and the reviewers. Any product that may be evaluated in this article, or claim that may be made by its manufacturer, is not guaranteed or endorsed by the publisher.
