# Supplementary material for: The Cerebrospinal Fluid Profile of Cholesterol Metabolites in Parkinson’s Disease and Their Association With Disease State and Clinical Features
Source: Front Aging Neurosci. 2021 Aug 30;13:685594. doi: 10.3389/fnagi.2021.685594 (PMC8435905; doi:10.3389/fnagi.2021.685594)
Supplement: Supplementary file 1 [file Table_1.DOCX]

Supplemental Figure 1. LC-MS(MS^n^) identification of 7α-hydroxy-27-*nor*cholest-4-ene-3,24-dione in CSF. (A) RIC of 539.4004 ± 5 ppm (upper panel) and 534.3690 ± 5 ppm (lower panel) and (B) MS^3^ (M^+^🡪M-Py^+^🡪) spectrum appropriate to 7α-hydroxy-27-*nor*cholest-4-ene-3,24-dione.

Supplemental Figure 2. Multivariate analysis on the data from Study 1 using SIMCA software and orthogonal projection to latent structures discriminant analysis (OPLS-DA). This yielded a robust model separating PD (blue) from controls (green) (Q2=0.68, ANOVA=3.2e-7 for cross-validated model).
